# Genomic insights from *Lactiplantibacillus plantarum* BRD3A isolated from Atingba, a traditional fermented rice-based beverage and analysis of its potential for probiotic and antimicrobial activity against Methicillin-resistant *Staphylococcus aureus*

**DOI:** 10.3389/fmicb.2024.1357818

**Published:** 2024-03-27

**Authors:** Surmani Huidrom, Ng Ngashangva, Joshua Khumlianlal, Kongbrailatpam Chandradev Sharma, Pulok Kumar Mukherjee, Sarangthem Indira Devi

**Affiliations:** ^1^Microbial Resources Division, Institute of Bioresources and Sustainable Development (IBSD), Imphal, Manipur, India; ^2^School of Biotechnology, Kalinga Institute of Industrial Technology (KIIT), Bhubaneswar, India

**Keywords:** fermented rice beverages, genome, *Lactobacillus*, probiotic, secondary metabolites, antimicrobial, MRSA

## Abstract

*Lactiplantibacillus plantarum* BRD3A was isolated from Atingba, a traditional fermented rice-based beverage of Manipur. Its genomic sequence has 13 contigs and its genome size is 3,320,817 bp with a guanine–cytosine (GC) ratio of 44.6%. It comprises 3185 genes including 3112 coding sequences (CDSs), 73 RNAs (including 66 tRNAs and others), and one clustered regularly interspaced short palindromic repeat (CRISPR) array. A comparative and phylogenetic analysis with the *Lp. plantarum* genome shows that this strain has close similarity with other *Lp. plantarum* strains and about 99% average nucleotide identity. Functional annotation using evolutionary genealogy of genes—non-supervised orthologous groups (EggNOG) and Kyoto Encyclopedia of Genes and Genomes (KEGG) reveals genes associated with various biological processes such as metabolism, genetic information processing, and transport functions. Furthermore, the strain harbors bacteriocins like plantaricin E, Plantaricin F, and Enterocin X categorized under class IIb by the BAGEL4 database, indicating its potential antimicrobial properties. Additionally, AntiSMASH web server predicted four secondary regions—T3PKS, terpene, cyclic lactone inducer, and ribosomally synthesized and post-translationally modified peptide (RiPP)—suggesting an even higher antimicrobial potential. We validated the antimicrobial activity of *Lp. plantarum* BRD3A through *in vitro* experiments in which it exhibited promising bactericidal effects on methicillin-resistant *Staphylococcus aureus*, inhibiting their biofilm growth. These findings indicate the potential of *Lp. plantarum* BRD3A to be used as an alternative to conventional antibiotics.

## 1 Introduction

*Lactiplantibacillus plantarum* is a lactic acid bacteria commonly found in fermented food and beverages and is generally recognized as safe (GRAS) by the U.S. Food and Drug Administration (FDA). It has also been approved for use by the European Food Safety Authority (Yilmaz et al., 2022). It is known as an antimicrobial, producing a diverse range of beneficial metabolites such as bacteriocins including modified bacteriocins such as lanthipeptides, cyclized peptides, sactipeptides, lasso peptides, and glycosylated bacteriocins as well as ribosomally synthesized and post-translationally modified peptides (RiPP)-like molecules (Huang et al., 2021; Montalbán-López et al., 2021). It also possesses probiotic properties which provide health benefits to humans. Many strains of *Lp. plantarum* isolated from different sources have both narrow and broad-spectrum antibacterial activity against Gram-positive and Gram-negative bacteria (Goel et al., 2020). *Lp. plantarum* has gained attention as it possesses the potential to be used in the development of new antimicrobial products, functional foods, and next-generation probiotics (Kandasamy et al., 2022).

Due to its methicillin resistance, methicillin-resistant *Staphylococcus aureus* (MRSA) causes adverse effects in humans as it confers resistance to penicillin and cephalosporins (Ali Alghamdi et al., 2023). It was categorized as a high-priority pathogen by the World Health Organization (WHO) in 2017 and causes approximately 150,000 hospital-associated infections each year, resulting in >7,000 deaths and a socioeconomic burden of EUR 380 million annually. So, alternative antibiotics to inhibit the growth of MRSA are urgently needed. MRSA is capable of forming a biofilm on biotic and abiotic surfaces, which enhances its antibiotic resistance and contributes to an increased rate of morbidity and mortality worldwide (Silva et al., 2021); therefore, effective therapeutic approaches are needed. Most of the previous studies suggested that lactic acid bacteria (LAB) strains can be used to eradicate biofilm-forming MRSA infections (Jalalifar et al., 2022).

The whole genome sequencing (WGS) analysis provides comprehensive genomic diversity and valuable insights for the prediction of safety assessment, such as identifying genes associated with antimicrobial resistance and virulence factors in LAB strains as recommended by the European Food Safety Authority (Chokesajjawatee et al., 2020). WGS also has the potential to uncover biosynthetic gene clusters (BCGs) in LAB using functional annotation prediction tools highlighting their ability to produce antimicrobials and enabling the characterization of probiotics based on their beneficial properties (Tenea and Ascanta, 2022).

Atingba, a traditional fermented rice-based beverage prepared and consumed by the Meitei and Kabui communities of Manipur in Northeast India has been part of the communities' ceremonial rituals for many generations. Traditionally, the beverage is valued for its medicinal properties in the treatment of ailments and diseases and possesses antimicrobial and antioxidant properties. Atingba is a non-distilled, mildly alcoholic beverage which is highly favored among the above mentioned communities and is prepared by fermenting cooked glutinous rice with the starter culture Hamei, associated with LAB involved in the fermentation process (Wahengbam et al., 2020). During fermentation, the predominant microbes found in Atingba consist of species from LAB including *Lactobacillus* and *Bifidobacterium* (Giri et al., 2018).

To the best of our knowledge, no studies have so far been done on the antimicrobial and probiotic characteristics of *Lactobacillus* isolated from Atingba, a traditional fermented rice-based beverage, based on genome sequencing, although previous studies have revealed its microbiota. This study employed a WGS approach to gain insights into *Lp. plantarum* BRD3A genome associated with metabolism, probiotic markers, biosynthetic gene clusters for antimicrobial peptide, and safety-relevant properties. *In vitro* studies of *Lp. plantarum* BRD3A were also carried out for determining its antibacterial and antibiofilm potential against MRSA. These findings help in the production of alternative antimicrobials to treat MRSA infections and potential probiotic strains.

## 2 Material and methods

### 2.1 Bacterial growth and DNA extraction

The strain BRD3A of *Lp. plantarum* was isolated from a fermented rice-based beverage (Atingba), which was previously identified based on 16S rDNA sequencing with GenBank accession no. MW031805. The strain was grown in De Man, Rogosa, and Sharpe (MRS) broth (Hi-Media, India) for 18 h at 37°C under anaerobic conditions. The cells were collected by centrifugation at 8,000 g for 5 min. Total genomic DNA was extracted from the cell pellets using a Genomic DNA Purification Kit (Thermo Scientific, USA) according to the manufacturer's instructions. The quantity and purity of DNA were checked spectrophotometrically at 260 nm using a NanoDrop ND-1000 UV-Vis Spectrophotometer (Thermo Scientific USA).

### 2.2 Genome sequencing, assembly, and annotation

The genomic DNA of the *Lp. plantarum* BRD3A strain was sequenced for identifying its WGS using the Illumina Nextseq 550 sequencing platform (2 × 151 paired ends). The quality of sequencing was estimated using FASTQC (version 0.12.0) (Andrews, 2010). For trimming of adapters and poor-quality sequence reads by paired sequence, Trim Galore (Version 0.6.5) (Krueger, 2015) was used and genome assembly was constructed by SPAdes (version 3.15.1) (Bankevich et al., 2012). Quality assessment of the assembled genome was carried out by QUAST (version 5.2) (Gurevich et al., 2013). The circular map was generated using the web server Proskee (Grant et al., 2023) for annotation and visualization.

### 2.3 Taxonomic classification and phylogeny

The genome sequence data were uploaded to the Type (Strain) Genome Server (TYGS) (https://tygs.dsmz.de/) to determine genome-based taxonomy using which genomic-scale phylogenies were identified. The estimate of species and subspecies for the determination of close-type genomic strains (Meier-Kolthoff and Göker, 2019) was done by calculating the pairwise digital DNA–DNA hybridization (dDDH) values between the user genome. The selected type of strains in the genome were identified using the Genome BLAST Distance Phylogeny (GBDP) formula d5. The tree was inferred using FastME 2.1.6.1 (Lefort et al., 2015) from GBDP distances calculated from genome sequences. The average nucleotide identity (ANI) with closely related species was determined using orthologous (Ortho) ANI values calculated from the OAT software (Lee et al., 2016) using reference genome strains.

### 2.4 Genomic and functional annotation

Genome annotation was carried out using an NCBI Prokaryotic Genome Annotation Pipeline (PGAP) (https://www.ncbi.nlm.nih.gov/genome/annotation_prok/) (Tatusova et al., 2016) and rapid annotations subsystems technology web server (RAST) (Aziz et al., 2008). The genome map was generated by CG Viewer (http://proksee.ca/) (Grant et al., 2023).

The functional annotations were carried out using the EggNOGmapper v2 (version 2.0) for the classification of predicted proteins into clusters of orthologous groups (COGs) (Cantalapiedra et al., 2021) and BlastKOALA (version 2.2) for the assignment of proteins into the Kyoto Encyclopedia of Genes and Genomes (KEGG) Orthology (KO) groups and the production of KEGG pathways (Kanehisa et al., 2016). The annotation of carbohydrate-active enzymes (CAZymes) was performed using the dbCAN2 meta server from CAZy database (Lombard et al., 2014; Zhang et al., 2018). The prediction of orthologous clusters by comparison with the available reference was performed using OrthoVenn 2 software (Xu et al., 2019).

### 2.5 Safety assessment for *Lp. plantarum* BRD3A

The genome of BRD3A was searched for antimicrobial resistance (AMR) genes by using the Resistance Gene identifier (RGI) tool of the Comprehensive Antibiotic Resistance Database (CARD) (Alcock et al., 2023) with default parameters such as Perfect and strict hits only and high quality/coverage. CRISPR CasFinder and PHAge Search Tool Enhanced Release (PHASTER) was used for the detection of clustered regularly interspaced short palindromic repeats (CRISPR), identification of Cas sequences, and annotation of prophage sequences (Arndt et al., 2016; Couvin et al., 2018).

### 2.6 Prediction of bacteriocins, secondary metabolites, and RiPP clusters

For the prediction of gene coding of bacteriocins and RiPPs, the BAGEL4 web server (http://bagel4.molgenrug.nl) was used by importing the contig FASTA sequences (van Heel et al., 2018). The National Center for Biotechnology Information's (NCBI) basic local alignment search tool (BLAST) was used to predict the core peptide databases (McGinnis and Madden, 2004) and AntiSMASH version 6.01 (Antibiotics and Secondary Metabolite Analysis SHell) (https://antismash.secondarymetabolites.org/) allows for the prediction of gene clusters encoding secondary metabolites (SMs) of all known broad chemical classes (Blin et al., 2021). The contigs FASTA file of the BRD3A was used as the input file, and default antiSMASH features were selected. For “KnownClusterBlast analysis,” the identified clusters were searched in the MIBiG database hits for the prediction of polyketides (PKs), nonribosomal peptide (NRPS), and RiPP-like peptide for the identification of putative secondary metabolites (Terlouw et al., 2023).

### 2.7 Growth kinetics studies and bacteriocin production

*Lp. plantarum* BRD3A strain was cultured in MRS broth incubated at 35±2°C without agitation for 48 h. The culture samples were then obtained at 4 h intervals for 48 h to monitor the changes in optical density (OD) at 600 nm (OD_600nm_), which was measured by spectrometer using an iMark Microplate Reader (Biorad, USA). The pH of the culture and antibacterial activity (Wang et al., 2018) were measured for bacteriocin production against the indicator MRSA strain ATCC 33591. The antibacterial activity was assessed by the agar-well diffusion method (Valgas et al., 2007) and the results were expressed as arbitrary units per milliliter of culture medium (AU/mL). One AU corresponds to the reciprocal of the highest 2–fold dilution showing a clear inhibition zone of the indicator strain (Deraz et al., 2005).

### 2.8 Antibacterial and antibiofilm activity of cell-free supernatant BRD3A against MRSA

#### 2.8.1 Preparation of cell-free supernatant

The cell-free supernatant (CFS) of *Lp. plantarum* BRD3A was collected from the growth culture obtained at 30 h of incubation at 37°C. It was recovered by centrifugation at 4000*g* for 15 min in 4°C followed by filtration using a cellulose acetate syringe filter of 0.22 μm pore size (Axiva Sichem Biotech, India). It was also used for further antibacterial and antibiofilm studies.

#### 2.8.2 Time kills assay

The MRSA strain ATCC 33591 was cultured overnight and diluted (1:100) with Tryptic soya broth (Hi-Media, India) and incubated at 37°C. The exponential growth phase of MRSA ATCC 33591 was incubated and treated with CFS BRD3A for 0, 2, 4, 6, 8, 10 and 12 h. The effect of CFS BRD3A on bacterial growth was evaluated without CFS BRD3A treatment as a control. It was aspirated out for serial dilution and plated on Tryptic Soya Agar (Hi-Media, India). The results were calculated and expressed as log CFU (colony-forming units)/ml count. Independent triplicate experiments were performed for the analysis (Sahoo et al., 2015).

#### 2.8.3 Antibiofilm activity

The overnight culture of MRSA ATCC 33591 was diluted 1:100 with fresh Tryptic soya broth (Hi-Media, India) culture medium, treated with CFS BRD3A, and without treatment for biofilm inhibition assay according to the previously reported method (Graziano et al., 2015). After incubating at 37°C for 24 h, the planktonic cells were gently washed twice with saline. The biofilm biomass was quantified with crystal violet staining. Briefly, 100 μl of 0.25% crystal violet (Hi-Media, India) was added to each well. After incubation at room temperature for 15 min, each well was washed using saline. Then, 100 μl of 95% ethanol (Hi-Media, India) was added to dissolve the stained dye for 20 min. The biofilm biomass was determined by measuring the absorbance at 570 nm with a microplate spectrophotometer iMark reader (BioRad, USA).

#### 2.8.4 Biofilm visualization in scanning electron microscopy (SEM)

The MRSA ATCC 33591 strain was used as a tested organism and cultured overnight in Tryptic soya broth containing 0.25% glucose (Hi-Media, India) supplemented with 50% CFS of BRD3A and then seeded onto the glass coverslips in a six-well plate (Costar, Cambridge, MA, USA). After static incubation at 37°C for 24 h, the biofilm was washed with phosphate buffer saline, fixed with 2.5% glutaraldehyde (Sigma, Germany) at 4°C overnight, and dehydrated in increasing concentrations (50%, 70%, 90%, and 100%) of ethanol (Yadav et al., 2015). The coverslips were fixed on aluminum stubs, covered with a gold–palladium film, and viewed under a field emission scanning electron microscope (FESEM) (JEOL JSM-IT800, USA).

### 2.9 Statistical analysis

All the experiments were carried out in triplicate. Statistical analysis was performed using Microsoft Excel (Microsoft 365) and GraphPad Prism (version 5) software.

## 3 Results

### 3.1 Genome assembly and features

**Figure 1:**
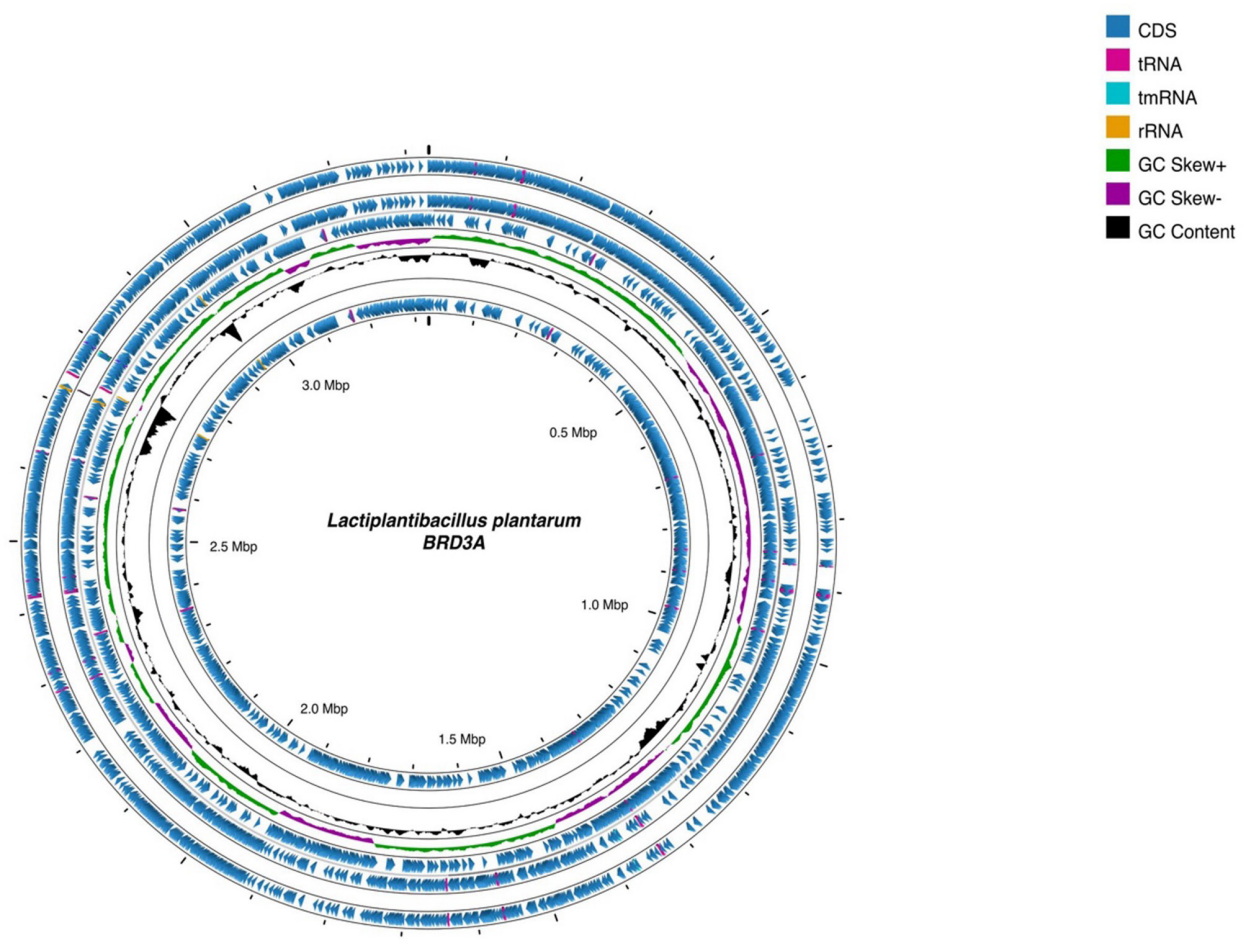
Circular genomic map of *Lactiplantibacillus plantarum* BRD3A using Proskee View server showing the characteristics features from outside to inner of the circle: CDS, RNA, GC skewness, and GC content are indicated in color code.

The whole genome sequencing of *Lp. plantarum* BRD3A strain isolated from the fermented rice beverage (Atingba) was performed using NextSeq 550 platform Illumina sequencing. The genome consists of 13 contigs and has an N50 value of 1,84,262 with a genome size of 3,308,953 bp and a guanine–cytosine (GC) ratio of 44.6%. Based on the PGAP and RAST for the genomic annotation, a total of 3,185 genes, including 3,112 protein coding sequence (CDS), 3,043 coding gene proteins, 73 RNAs, 66 tRNAs, 49 miscRNAs, 3 rRNAs, 1 tmRNA, and 1 CRISPR array were predicted. The circular genomic map of *Lp. plantarum* BRD3A was annotated and visualized by Proskee, which showed the characteristics of the genome such as the distribution of the genes on the forward and antisense strands, functional classification of genes, and GC content, as shown in Figure 1.

### 3.2 Taxonomic classification and phylogenetic analysis

**Figure 2:**
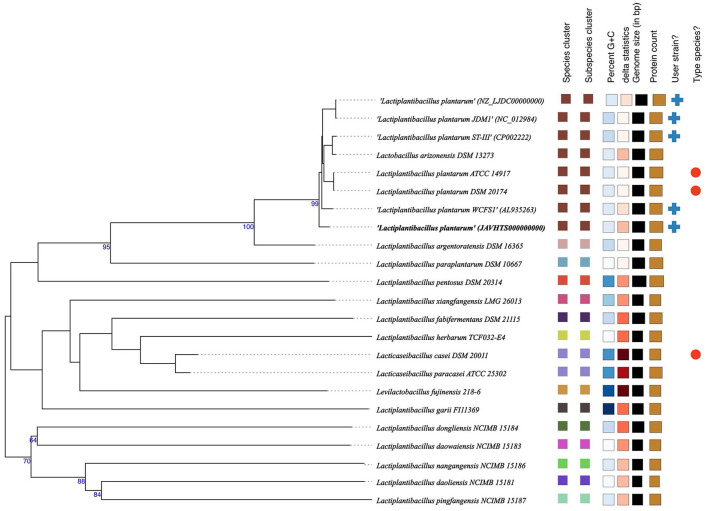
Phylogenetic comparisons of *Lactiplantibacillus plantarum* BRD3A with representative genomes of other reference strains carried out in the TYGS webserver indicate *Lp. plantarum* WCFS1 was the closest strain. The tree was inferred with FastME 2.1.6.1 from GBDP distances calculated from genome sequences. The branch lengths are scaled in terms of GBDP distance. The numbers above branches are GBDP pseudo-bootstrap support values > 60% from 100 replications.

**Figure 3:**
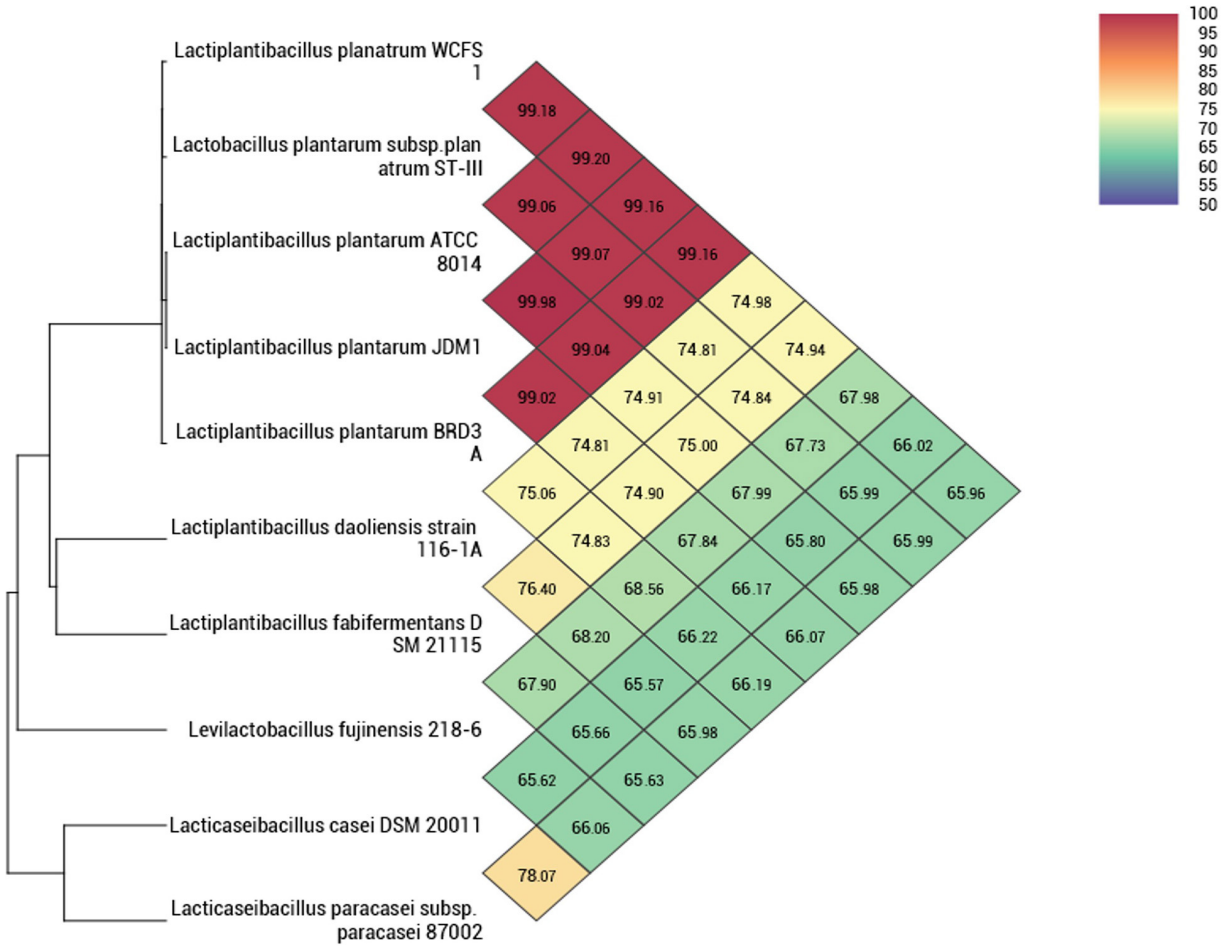
Heat map of OrthoANI of *Lp. plantarum* BRD3A compared with closely related *Lp. plantarum* strains that show 99% similarity with *Lp. plantarum* WCFS1, *Lp. plantarum* ST-IlI, and *Lp. plantarum* ATCC 8014.

The whole genome taxonomy of the *Lp. plantarum* BRD3A strain was compared with the closest types of 21 strain genomes available in the TYGS database based on the phylogenomic analysis using genome–genome taxonomy, which revealed that the *Lp. plantarum* BRD3A strain showed similarity with other strains such as *Lp. plantarum* DSM 20174 and *Lp. plantarum* ATCC 14917, while *Lp. plantarum* WCFS1 showed a close distance (Figure 2). Therefore, the results of the TYGS analysis confirmed the identification of the genes. The ANI-based taxonomy was analyzed based on orthoANI values, which revealed that the strain BRD3A had 78.06–99.18% genome sequence similarities with the closely related species shown in Figure 3 with a high percentage of similarity with *Lp. plantarum* ATCC 8014 (99.22%) followed by *Lp. plantarum* WCFS1 (99.20%), *Lp. plantarum* EM (99.16%), and *Lp. plantarum* STIII (99.03%), which indicate that the BRD3A strain belongs to *Lp. plantarum* species.

### 3.3. Functional characterization and annotation

The functional annotated genes predicted by RAST for *Lp. plantarum* BRD3A indicate the total number of 1,985 CDS were distributed in 339 seed culture expansion process (SEED) subsystems and prediction for the 43% coverage subsystems revealed a total of 1,334 features, which include 1,274 non-hypothetical subsystems and 60 hypothetical subsystems. The carbohydrate subsystem category consists of the highest proportion (17.3%) followed by amino acid derivatives (12.1%), protein metabolism (11.2%), cofactors, vitamins, prosthetic groups, pigments (7.2%), RNA metabolism (7.1%), and cell wall and capsule (6.6%), as shown in Table 1.

**Table 1:** Biological subsystem distribution of the genes by RAST annotation for the genome *Lp. plantarum* BRD3A.

**Description**	**Value**	**Percent**
Amino acids and derivatives	173	12.1
Cell wall and capsule	95	6.6
Virulence, disease and defense	37	2.5
Respiration	15	1.0
Fatty acids, lipids, and isoprenoids	64	4.4
Sulfur metabolism	12	0.8
Nucleosides and nucleotides	80	5.6
Secondary metabolism	4	0.3
Phages, prophages, transposable elements, plasmids	15	1.0
Dormancy and sporulation	6	0.4
Dna metabolism	85	5.9
Cofactors, vitamins, prosthetic groups, pigments	104	7.2
Stress response	46	3.2
Phosphorus metabolism	27	1.8
Membrane transport	49	3.4
Carbohydrates	247	17.3
Metabolism of aromatic compounds	5	0.3
Protein metabolism	160	11.2
Potassium metabolism	12	0.8
RNA metabolism	102	7.1
Regulation and cell signaling	26	1.8
Cell division and cell cycle	40	2.8

**Figure 4:**
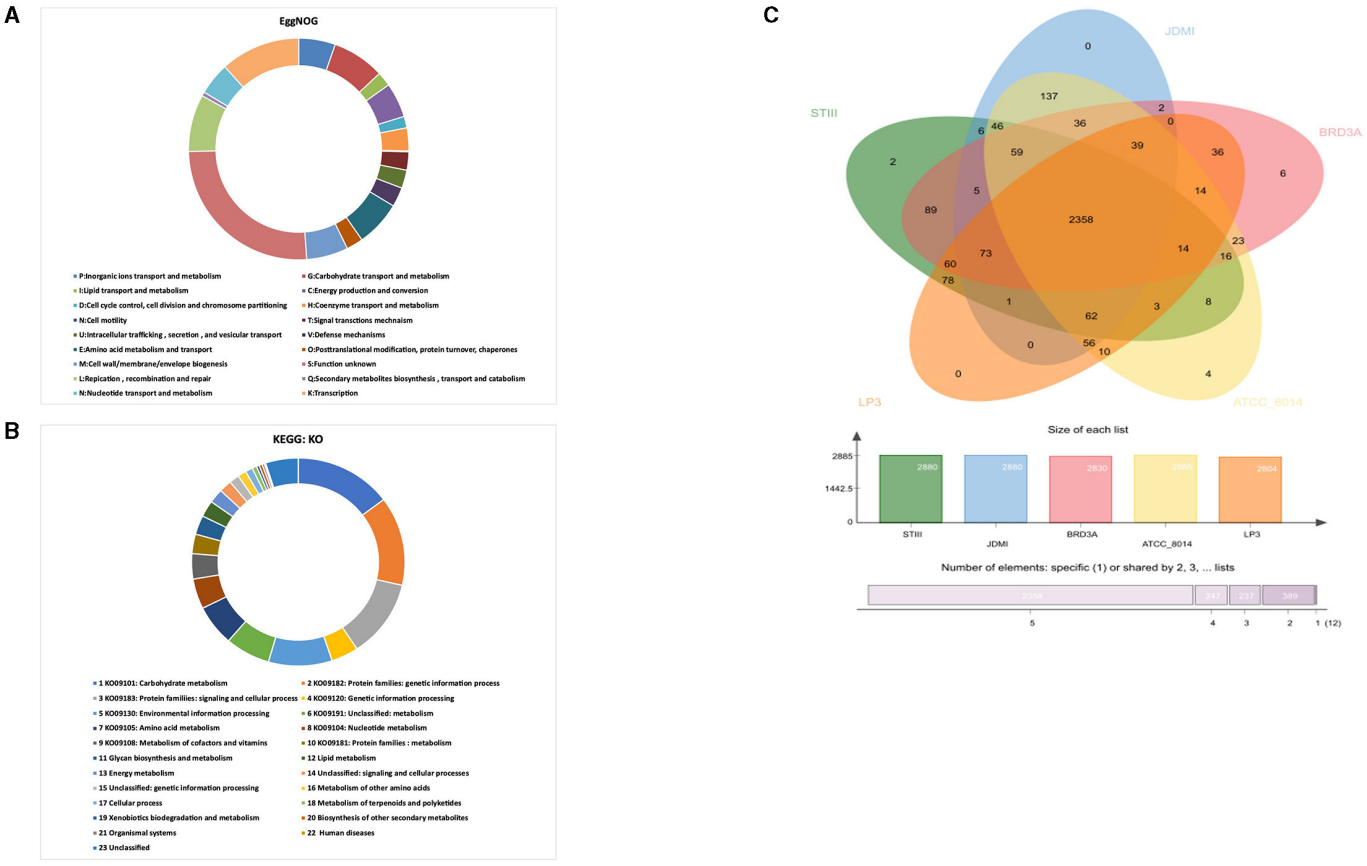
**(A)** EggNOG prediction of Cluster of Orthologous group (COG) functional categories to the proteins BRD3A strain, **(B)** KEGG orthology (KO) categories of identified protein-coding genes in the *Lp. plantarum* BRD3A, and **(C)** OrthoVenn diagram indicates the distribution of common genes (orthologous clusters) among *Lp. plantarum* STIII, *Lp. plantarum* JDM1, *Lp. plantarum* LP3, *Lp. plantarum* ATCC 8014, and *Lp. plantarum* BRD3A.

The clusters of orthologous groups (COG) revealed by the evolutionary genealogy of genes—non-supervised orthologous groups (EggNOG) mapper v2 annotated 2,326 genes comprising 18 functional categories (Figure 4A). The genes are classified into functional categories for carbohydrate transport and metabolism (G):179, inorganic ions transport and metabolism (P):125, lipid transport and metabolism (L):49, energy production and conversion (C):118, cell cycle control, cell division and chromosome partitioning (D):39, coenzyme transport and metabolism (H):78, signal transduction mechanism (T):62, intracellular trafficking, secretion, and vesicular transport (U):62, defense mechanisms (V):66, amino acid metabolism and transport (E):156, posttranslational modification, protein turnover, chaperones (O):57, cell wall/membrane/envelope biogenesis (M):142, replication, recombination and repair (L):194, secondary metabolites biosynthesis, transport, and catabolism (Q):14, nucleotide transport and metabolism (N):110, and transcription (K):272. The highest proportion of genes was present under function unknown (S):600 while cell motility (N):3 was associated with a small proportion of genes.

Furthermore, BlastKOALA revealed that a total of 1,451 annotated genes were classified into 23 KEGG functional categories in *Lp. plantarum* BRD3A (Figure 4B). It suggested that the highest number of genes encoding proteins (214) are responsible for carbohydrate metabolism (KO09101), followed by others for protein families: genetic information process (KO09182) (201), protein families: signaling and cellular processing (KO09120) (176), and environmental information processing (KO00130) (140).

The OrthoVenn diagram analysis revealed that the core genome contains 2358 orthologous proteins, which were compared with four closely related strains, and it revealed the species contains 3,243 clusters, 3,242 orthologous clusters (at least two species), and one-single copy gene cluster. The BRD3A strains share the orthologous proteins with *Lp. plantarum* STIII, *Lp. plantarum* JDM1, *Lp. plantarum* LP3, and *Lp. plantarum* ATCC 8014, as shown in Figure 4C.

### 3.4 Prediction of carbohydrate-active enzymes

The prediction of carbohydrate-active enzymes for BRD3A genome by the dbCAN web server identified five major classes in the CAZy database and predicted a total of 87 genes, which are classified under glycoside hydrolase (GH) (46) genes, glycosyltransferase (GT) (33) genes, carbohydrate esterase (CE) (4) genes, carbohydrate-binding modules (CBMs) (3), and auxiliary activity (AA) (3) genes. The highest number of CAZy genes found in GHs and GTs in the BRD3A strain were classified into different families, which suggests that it has potential probiotic activity in the defense against pathogens and immune stimulation.

### 3.5 Presence of probiotic-related genes

The *Lp. plantarum* BRD3A genome harbors probiotic-related genes responsible for proteins coding genes involved in stress response (temperature, bile, pH) and adhesion genes, which includes cold-shock protein (cspA) that contains three genes related to survival under low temperatures. Heat-shock proteins encode 16 genes, which include molecular chaperones (*dna*K, *dna*J, *hsl*O, *Grp*E, *gros*ES, *gros*EL, *htp*X) and protease encoding genes (*Clp*E, *Clp*P, *Clp*X, *Clp*C, *hsl*V, *hsl*U). For acid tolerance, there are genes (*atp*C, *atp*D, *atp*G, *atp*A, *atp*H, *atp*F, *atp*E, *atp*B, and *gad*B) encoding resistance in low pH conditions and bile stress, which include three encoding proteins (choloylglycine hydrolase) and adhesion-related genes (*esp*A, *esp*B, *gpr, tuf* , *srt*A, and enolase), which indicates the strain's high adhesion ability, as shown in Table 2. These results suggest that the BRD3A strain might resist multiple stress conditions and be consistent with the adaptability characteristics of the gastrointestinal tract.

**Table 2:** Annotation of probiotic-related genes identified in the *Lp. plantarum* BRD3A genome.

**Gene**	**Function**	**No of Gene**
**Temperature**		
ClpE	ATP-dependent Clp protease ATP-binding subunit ClpE	1
ClpP	ATP-dependent Clp protease, protease subunit [EC:3.4.21.92]	1
HSP20	HSP20 family protein	1
ClpL	ATP-dependent Clp protease ATP-binding subunit ClpL	1
HSP90	Heat-shock protein	1
DnaK	Heat-shock 70 kDa protein	1
dnaJ	Molecular Chaperone DnaJ	1
clpX	ATP-dependent Clp protease ATP-binding subunit ClpX	1
GrpE	Molecular chaperone GrpE	1
hslO	molecular chaperone Hsp33	1
hslV	ATP-dependent HslUV protease, peptidase subunit HslV [EC:3.4.25.2]	1
hslU	ATP-dependent HslUV protease ATP-binding subunit HslU	1
htpX	Heat-shock protein HtpX [EC: 3.4.24.-]	1
ClpC	ATP-dependent Clp protease ATP-binding subunit ClpC	1
groES	Chaperonin GroES	1
groEL	Chaperonin GroEL [EC:5.6.1.7]	1
CspA	Cold-shock protein	3
**Acid tolerance**		
atpC	F-type H+-transporting ATPase subunit epsilon	1
atpD	F-type H+/Na+-transporting ATPase subunit beta [EC:7.1.2.2 7.2.2.1]	1
atpG	F-type H+-transporting ATPase subunit gamma	1
atpA	F-type H+/Na+-transporting ATPase subunit alpha [EC:7.1.2.2 7.2.2.1]	1
atpH	F-type H+-transporting ATPase subunit delta	1
atpF	F-type H+-transporting ATPase subunit b	1
atpE	F-type H+-transporting ATPase subunit c	1
atpB	F-type H+-transporting ATPase subunit a	1
gadB	gadB, gadA, GAD; glutamate decarboxylase [EC:4.1.1.15]	1
**Bile tolerance**		
Pva1	Linear amide C–N hydrolase, choloylglycine hydrolase family protein	1
Yxel	Linear amide C–N hydrolase, choloylglycine hydrolase family protein	1
Cbh	Choloylglycine hydrolase [EC:3.5.1.24]	1
**Adhesion**		
espA	Protein–tyrosine kinase modulator	1
espB	Protein–tyrosine kinase [EC:2.7.10.3]	1
gpr	L-glyceraldehyde 3-phosphate reductase [EC:1.1.1.-]	1
tuf	Elongation factor Tu	1
srtA	Sortase A [EC:3.4.22.70]	1
ENO	Enolase [EC:4.2.1.11]	1

### 3.6 Safety assessment of the *Lp. plantarum* BRD3A strain

The BRD3A genome predicted from the CARD database revealed two strict hits: (i) *van*Y coding gene (glycopeptide antibiotic) with 31.33% identity and (ii) *qac*J, which gives it small multidrug resistance (SMR), leading to its classification in the drug class as a disinfecting agent and an antiseptic with 40.2% identity of the matching regions (Supplementary Figure 1).

Two CRISPR arrays were identified in the *Lp. plantarum* BRD3A, one of which is in contig BRD3A 13_1 (start 1089941-end1090028) with 28 bp repeat matching a consensus sequence with evidence level 1 with 87 CRISPR length and BRD3A 13_2 (start 1154062-end 1154427) with 36 bp repeat length evidence level 4 with 365 CRISPR length. The presence of CRISPR/CRISPR-associated (Cas) systems was Type II-A that includes cas 9, cas 1, cas 2, and csn 2, which were identified a CRISPRCasFinder analysis (Tables 3, 4) and annotated by COG and RAST.

**Table 3:** *Lp. plantarum* BRD3A strain CRISPR array system using CRISPRCasFinder.

**CRISPR ID**	**Start**	**End**	**Consensus repeat**	**No.of CRISPRs with same repeat**	**Repeat length**	**No.of spacers**	**Evidence level**	**Length (bp)**	**Orientation**
BRD3A-13-1	1089941	1090028	TTTTTTCCGATTTTTTCATAAAAATCTT	0	28	1	1	87	unknown
BRD3A-13-2	1154062	1154427	GTCTTGAATAGTAGTCATATCAAACAGGTTTAGAAC	4	36	5	4	365	Forward

**Table 4:** *Lp. plantarum* BRD3A strain Cas type detection using CRISPRCasFinder.

**CAS-TypeIIA**	**Gene status**	**Start**	**End**	**Orientation**
cas9_TypeII	Mandatory	1,147,904	1,151,980	+
cas1_TypeII	Accessory	1,152,175	1,153,080	+
cas2_TypeI-II-III	Accessory	1,153,058	1,153,363	+
csn2_TypeIIA	Mandatory	1,153,360	1,154,037	+

**Figure 5:**
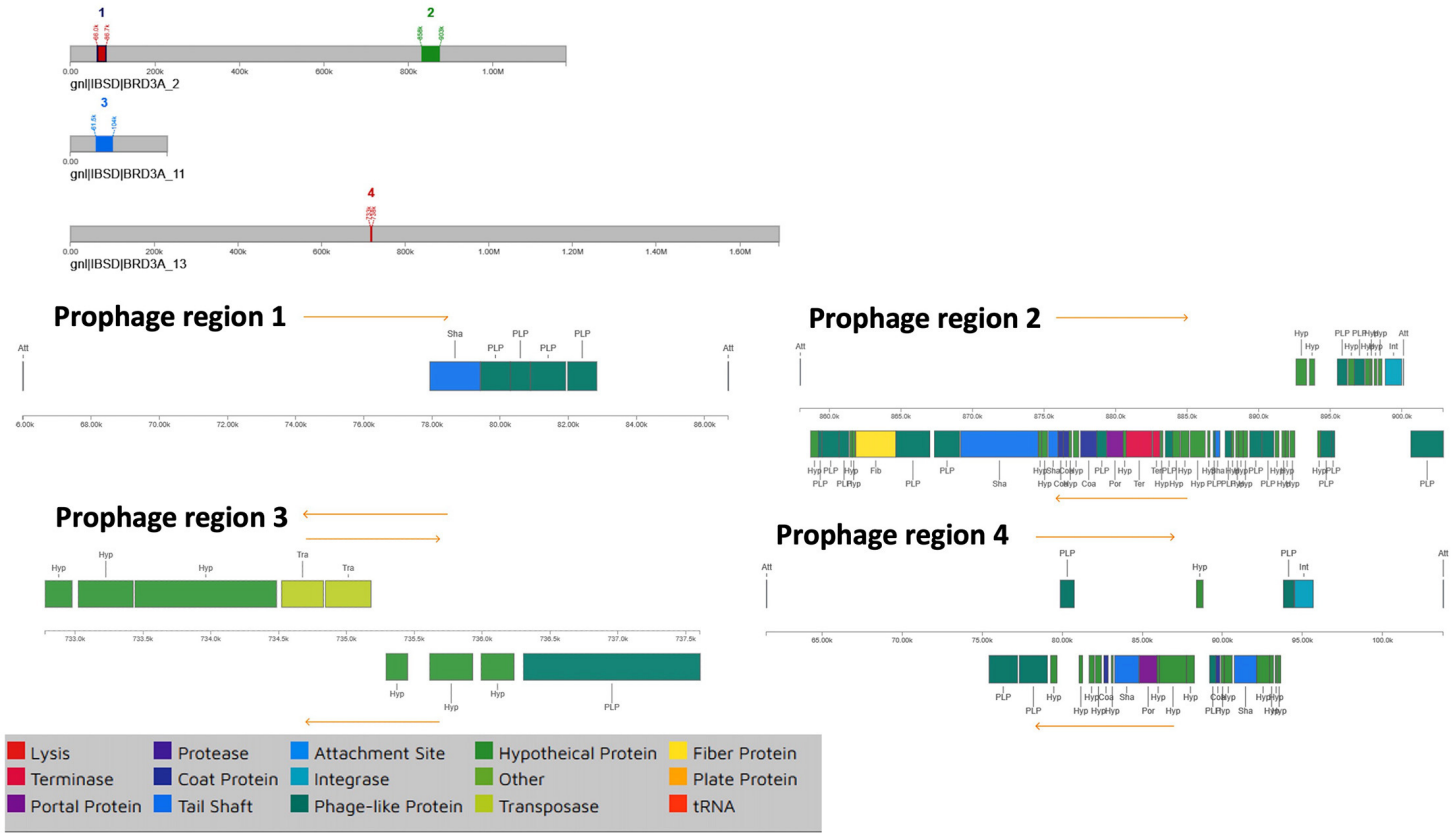
Prophage regions by PHASTER which shows all the regions including the 4 prophage regions in the BRD3A genome which shows one intact, two incomplete and one questionable.

The BRD3A strain genome of the prophage sequence using PHASTER revealed four prophage regions, comprising one intact, two incomplete, and one questionable regions (Figure 5). The sizes of these regions were 20.6kb, 44.9kb, 42.2kb, and 4.8 kb, respectively. The intact prophage (Region 2) exhibited the highest protein count of 56 with a GC content of 40.57% with the most common:PHAGE Lactob_Sha1_NC_019489(16). In the prophage (Region 3), the questionable region corresponded to PHAGE_Staphy_SA7_NC_048658 with a GC proportion of 43% containing 26 proteins. The incomplete prophage was detected within the region 1 PHAGE_Escher_vB_EcoM_Schickermooser_NC_048196(4) with seven proteins and region 4 PHAGE_Lactob_Lb_NC_047983(2) with nine proteins. The phage attachment sites (attL/attR) in Regions 2 and 4 were located upstream of the integrase, which are useful as markers for mobile DNA elements as shown in Table 5.

**Table 5:** Prophage regions of *Lp. plantarum* BRD3A identified in PHASTER.

**Region**	**Region length**	**Completeness**	**Score**	**Total proteins**	**Region position**	**Most common phage**	**GC%**	**attL/attR sites**
1	20.6Kb	Incomplete	20	7	65995-86685	PHAGE_Escher_vB_EcoM_Schickermooser_NC_048196(4)	36.99%	yes
2	44.9Kb	Intact	150	56	857975-902900	PHAGE_Lactob_Sha1_NC_019489(16)	40.57%	yes
3	42.2Kb	Questionable	80	26	61536-103792	PHAGE_Staphy_SA7_NC_048658(2)	43.27%	yes
4	4.8Kb	Incomplete	30	9	732781-737607	PHAGE_Lactob_Lb_NC_047983(2)	42.24%	no

### 3.7 Prediction of biosynthetic gene clusters for bacteriocin

**Figure 6:**
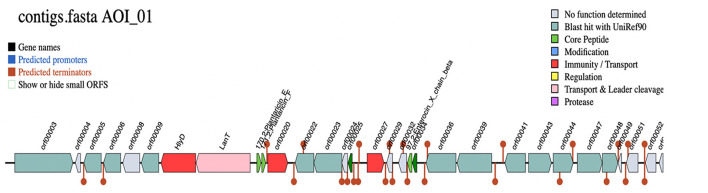
Biosynthetic gene clusters (BCGs) for *Lp. plantarum* BRD3A HlyD-Accessory factor for PInH, LanT-Bacteriocin ABC-transporter, ATP-binding, and permease protein PInG. Plantaricin EPlantaricin F: gg motif; Lactococcin; Bacteriocin_IIc; 171.2; Plantaricin_F, Enterocin_^*^_chain_beta-ComC; Lactococcin; Bacteriocin_IIc; 97.2; Enterocin_X _chain beta.

The biosynthetic gene clusters (BGC) for the production of bacteriocin were predicted by the BAGEL4 web server, which identified one area of interest (AOI) within the *Lp. plantarum* BRD3A genome. The AOI for the BRD3A located within contig (2.7) encodes the bacteriocin Plantaricin such as PlnE (Bit score: 112.464, Evalue: 3.77e), PlnF Lactococcin; Bacteriocin_IIc; (Bit score:107.071, E-value: 3.59e-36) and Enterocin X as ComC; Lactococcin; Bacteriocin_IIc; 97.2; Enterocin_X_chain_beta (Bit score: 50.8322, Evalue:1.32e-13), which is lactoccin-like family with double glycine leader peptide similar to chain beta-protein sequence as core peptides, which implies that the *Lp. plantarum* BRD3A might be a producer of class II bacteriocins. Additionally, HlyD bacteriocin is an accessory factor for ABC transporter PlnH (LanT) and Bacteriocin ABC transporter, adenosine triphosphate (ATP)-binding and permease protein PlnG, and PlnI bacteriocin immunity protein, which belongs to membrane-bound protease CAAX family (bit score = 99.216%). The additional open reading frames (ORFs) predicted the presence of putative bacteriocin immunity protein found in the same bacteriocin gene clusters and PlnD response regulator and a repressor for transcription factor and bacteriocin production-related histidine kinase within ORFs, as shown in Figure 6, Supplementary Table 1.

#### 3.7.1 Production of secondary metabolites and RiPP

**Figure 7:**
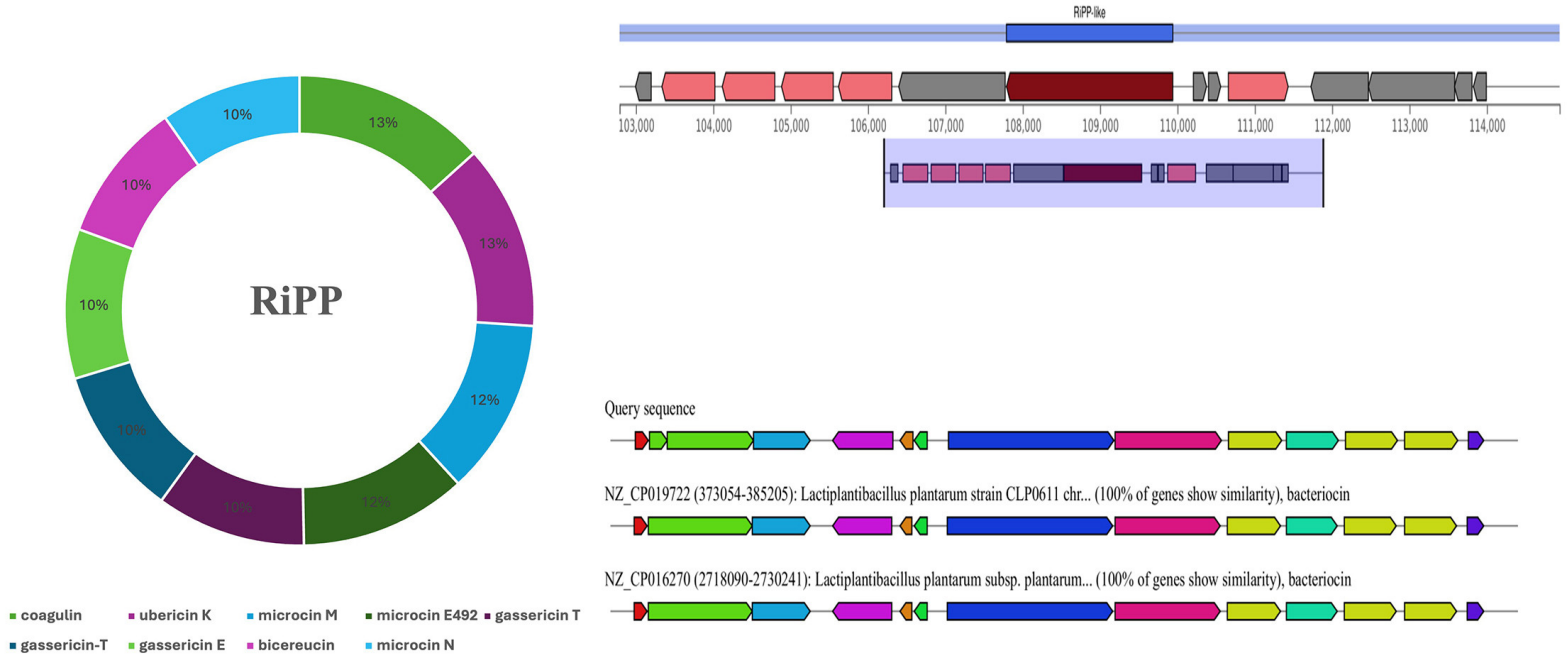
Secondary metabolites prediction in the *Lp. plantarum* BRD3A genome by antiSMASH showing RiPP metabolites (Coagulin) highest score similarity by MIBiG database.

The prediction of secondary metabolites and RiPP by using antiSMASH revealed four regions in BRD3A-2 Region 1, type III polyketide synthase (T3PKS) (total: 41,170 nt); BRD3A-2 Region 2, RiPP-like peptides (total: 12,151 nt); BRD3A 13 region 1, terpene (total: 20,882 nt); and BRD3A 13 region 2, cyclic lactone autoinducer (total: 20,706 nt). The prediction of metabolites and similarity scores were done by a comparison with the MiBIG database of several gene clusters that were detected (Supplementary Table 1), which include RiPP with 42.55%, the highest in number. The prediction MIBiG showed Enterocin F4-9 (*Enterococcus faecalis*) and Coagulin (*Bacillus coagulans*) in RiPP having the highest similarity score. The KnownClusterBlast for RiPP in BRD3A showed that coagulin has similar gene clusters in *Lp. plantarum* CLP0611 (Figure 7).

### 3.8 Kinetic studies of bacteriocin production in *Lp. plantarum* BRD3A

**Figure 8:**
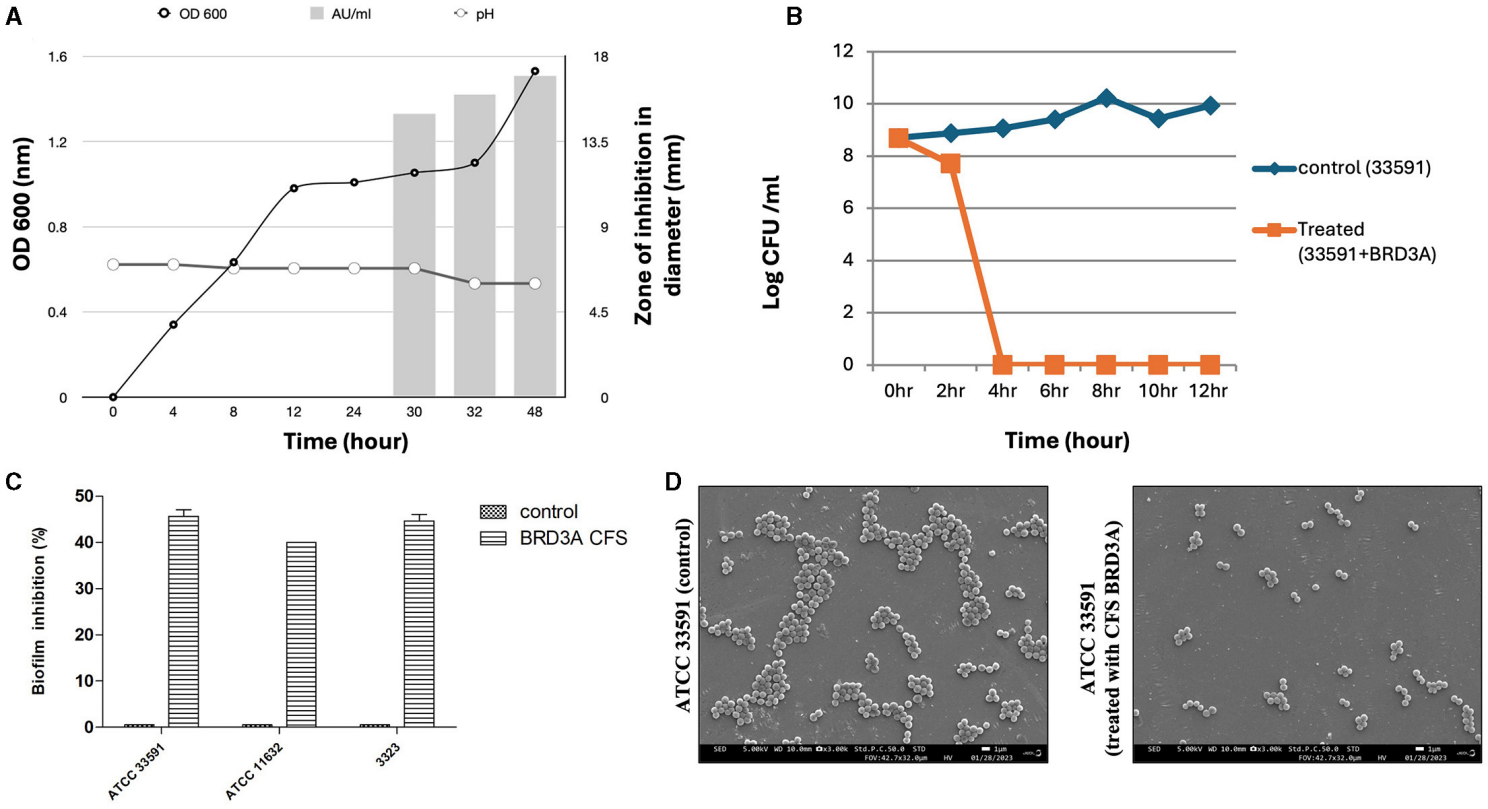
**(A)** Growth kinetics and dynamics studies of bacteriocin production at different time intervals, **(B)** Time kill studies of Methicillin-resistant *Staphylococcus aureus* ATCC 33591 showing bactericidal effect after 4 h treatment with BRDA, **(C)** Biofilm inhibition of BRD3A against MRSA, and **(D)** Field emission SEM image in (3.00X) magnification shows MRSA untreated biofilm (control) and when MRSA biofilm treated with BRD3A it showed disruption of biofilm matrix.

The *Lp. plantarum* BRD3A strain showed that a stationary growth phase after incubation for 24–32 h and a decrease in the pH value of the medium. The production of bacteriocin in BRD3A started at 30 h (growth phase at pH 6), showing a bacteriocin activity (1000 AU/ml) against indicator strain MRSA ATCC 33591, which was indicated by the diameter of zone inhibition (Figure 8A).

### 3.9 Antibacterial and antibiofilm activity

Time-kill assay indicated that CFS BRD3A demonstrated a significant reduction in the value of log CFU/ml at 4 h for MRSA ATCC 33591 (8 log CFU/ml), demonstrating a bactericidal effect, which indicates antibacterial activity (Figure 8B). Crystal violet staining assay exhibited the activity of CFS BRDA against biofilm formation of MRSA ATCC 33591, *Staphylococcus aureus* ATCC 11632, and MRSA clinical strain MRSA at an inhibition percentage of 46%, 38%, and 45% respectively (Figure 8C). The FESEM analysis visualization of MRSA ATCC 33591 and the biofilm cells treated and untreated with the BRD3A CFS revealed a reduction in the number of the biofilm cells, demonstrating its potential antibiofilm activity (Figure 8D).

## 4 Discussion

In this study, the whole genome sequence analysis of *Lp. plantarum* BRD3A strain isolated from Atingba, a fermented rice beverage, revealed a genome size of 3,308,953 bp and a GC ratio of 44.6%. The genome size and GC ratio of *Lp. plantarum* BRD3A showed similarity with the previously reported *Lp. plantarum* strains isolated from different sources. The larger genome size and GC ratio of *Lactobacillus* strains mean it reflects the environmental adaptability of the strains (Li et al., 2023). The genome-based taxonomy by TYGS revealed that it is closely related to *Lp. plantarum* DSM 20174 and *Lp. plantarum* ATCC 14917. The orthoANI values of the BRD3A genome showed 99% similarity with the reference strains *Lp. plantarum* WCFS1, *Lp*. *plantarum* EM, and *Lp. plantarum* STIII, which confirmed that this strain belongs to the species *Lp. plantarum*.

Furthermore, the prediction of functional annotation by RAST and COG for *Lp. plantarum* indicated a unique potential with a total number of 1,985 CDS distributed in 339 SEED subsystems and COG 2,326 genes classified into 18 functional categories, with the highest number of genes under function unknown. The larger number of unknown function genes reveals the uniqueness and unknown potential of the strain. The BlastKOALA analysis revealed that a total of 1,555 functional categories were classified into 23 KEGG pathways in *Lp. plantarum* BRD3A. These findings of the functional annotation analysis revealed that the BRD3A strain has high metabolic capacity with a high number of genes associated with carbohydrate metabolism and transport followed by genetic information processing, which indicate their importance in conserved cellular processes in this genome. Lactic acid bacteria are reported to be rich in carbohydrate metabolism genes and can use a variety of carbohydrates, which might also provide energy for growth (Ghattargi et al., 2018). The BRD3A core genomes comprised 2358 orthologous proteins, which are essential genes. They are highly conserved within most of the *Lp. plantarum* and play an important role in cell cycle, carbohydrate, and protein metabolism (Huang et al., 2020).

In the BRD3A genome, there were 87 genes belonging to five major classes of CAZymes, with the highest number of genes found in clusters belonging to the glycoside hydrolase family. These enzymes play a role in synthesizing, modifying, and disintegrating complex carbohydrates and glycoconjugates found abundantly in LAB (Kandasamy et al., 2022). Research indicates that GH family genes play a significant role in diverse biological processes such as sugar biosynthesis, cellular metabolism, and signaling (Zhao et al., 2023).

The genome of BRD3A consists of probiotic-related genes involved in heat stress, cold stress, acid tolerance, bile tolerance, and adhesion. These results suggest that the strain might resist multiple stress conditions and be consistent with the adaptability characteristics of the gastrointestinal tract. According to a previous study by Kandasamy et al. (2022), many lactobacilli strains harbor a huge number of probiotic gene-encoding proteins involved in stress responses.

The BRD3A genome predicted from the safety assessment based on the CARD database shows *van*Y coding gene (glycopeptide antibiotic), which indicates that most of the strains of *Lp. plantarum* BRD3A are resistant to vancomycin due to their intrinsic resistance mechanism, and the presence of *qac*J conferring it with small multidrug resistance (SMR), leading to its classification in the drug class as a disinfecting agent and an antiseptic. It is common for *Lp. plantarum* strains to exhibit intrinsic resistance to vancomycin (Gueimonde et al., 2013).

Our findings revealed that the BRD3A harbored two CRISPR arrays and the presence of CRISPR/CRISPR-associated (Cas) systems belonging to Type II-A, including cas 9, cas 1, cas 2, and csn, pointing out that the strain plays a significant role in defending the organism against foreign genetic elements. The CRISPR/Cas system serves as an adaptive defense mechanism to prevent the strains from developing resistance to antimicrobials and pathogenicity through horizontal gene transfer (Rodrigo-Torres et al., 2019).

Prophages are often common in the probiotic *Lactobacillus* species as a genetic component (Zhang et al., 2019). The BRD3A genome predicted four prophages—one intact, two incomplete, and one questionable—with a length of 44 kb intact regions. This indicates its potential effectiveness against invasion by other prophages (Tenea and Ortega, 2021). Two integrases were identified in the region of prophage 2 and prophage 4. Most integrase bacterial genomes are useful markers for prophages for DNA elements (Zhang et al., 2019).

The biosynthetic gene clusters (BGC) for bacteriocin production encode core peptides PlnE, plnF, and Enteroxin X beta chain in the genome of BRD3A. This core peptide Plantaricin plnE/F was found in *Lp. plantarum strains* reported by Goel et al. (2020) such as in DHCU70 from (dahi), DKP1 (kinema), fermented soybean (Rodrigo-Torres et al., 2019), and CECT 8,963 from Chicha, a traditional maize-based fermented beverage. Tenea and Ortega (2021) reported that *Lp. plantarum* UTNGt2 from white cocoa had the presence of both plantaricin pln E/F and Enterocin X beta chain as biosynthetic gene clusters. EnterocinX is a novel dual-peptide heat-resistant bacteriocin composed of two antimicrobial peptides (Xα and Xβ), the complementarity of which enhances its antibacterial activity (Hu et al., 2010). It implies that the *Lp. plantarum* BRD3A might be a producer of bacteriocin belonging to class IIb bacteriocins, which possess bacteriogenic activity against the Gram-positive and Gram-negative bacteria.

In the present study, the secondary metabolites of BRD3A had four metabolite regions, which include T3PKS, RiPP-like, terpene, and cyclic lactone autoinducer. Among them, the RiPP-like region consists of major biosynthetic gene clusters. The MiBIG database for RiPP-like region revealed the highest similarity percentage as exhibited in coagulin, which is common in most of the *Lp. plantarum* strains reported from the previous studies (Tenea and Ortega, 2021; Tenea and Ascanta, 2022).

The production of bacteriocin by the BRD3A strain against indicator strain MRSA ATCC 33591 was observed at 30 h in the late exponential phase under pH 6. The bacteriocin activity was expressed at 1,000 AU/ml. Our findings from the time-kill assay revealed a significant reduction in the value of log CFU/ml after treatment with CFS BRD3A, indicating its bactericidal effect on MRSA ATCC 33591 within 4 h of incubation. Furthermore, CFS BRD3A exhibited good antibiofilm against MRSA ATCC 33591, *Staphylococcus aureus* ATCC 11632, and MRSA clinical strain, showing effective biofilm inhibition of 46%, 38% and 45%, respectively, when analyzed by crystal violet stain method. Visualizing the biofilm matrix using scanning electron microscopy (SEM) confirmed that treatment with CFS BRD3A resulted in a substantial 50% reduction and disruption of the biofilm matrix of MRSA. Few studies have also shown that lactic acid bacteria such as *Lactobacillus* species exhibit significant antibacterial and antibiofilm activity against MRSA (Jalalifar et al., 2022). Therefore, it can be concluded that CFS BRD3A demonstrated antibacterial properties along with biofilm inhibitory capabilities, which make it an effective therapeutic option against *S. aureus* infections.

## 5 Conclusions

Overall, the study showed that the whole genome characterization of *Lp. plantarum* strain BRD3A strain, based on our knowledge, is the first genomic study *of Lp. plantarum* isolated from Atingba, a traditional fermented rice-based beverage commonly used for traditional ritual purposes and as ethnomedicine properties for various ailments and diseases by the ethnic tribes of Manipur. Genomic characterization and *in silico* approach revealed various protein families, metabolisms, and harboring genes encoding for various secondary metabolites (SMs), BGCs, RiPP molecules, NRPSs, and terpenes, which confer the strain with antimicrobial properties. The BRD3A genome revealed various associated probiotic-related genes such as stress adaptation to the gastrointestinal tract and cell adhesion, which make it a potential probiotic candidate. For safety concerns, genomic stability was confirmed by the presence of prophage regions and the CRISPR-Cas system, which prevents the strain from acquiring resistance to genes (virulence and antibiotics), and the absence of plasmid. *In vitro* studies showed that the BRD3A strain has a bactericidal effect against MRSA and exhibited inhibition of the biofilm matrix of MRSA strain, which clearly shows that *Lp. plantarum* strain BRD3A possesses potential antibacterial and antibiofilm activity due to the presence of antimicrobial metabolites and can be used as an alternative to conventional antibiotics and in the development of novel therapeutics for health benefits.

## Data availability statement

The datasets presented in this study can be found in online repositories. The names of the repository/repositories and accession number(s) can be found in the article/Supplementary material.

## Author contributions

SH: Formal analysis, Investigation, Visualization, Writing – original draft, Methodology, Software, Conceptualization. NN: Software, Visualization, Writing – review & editing, Formal analysis, Validation, Methodology. JK: Formal analysis, Investigation, Writing – review & editing. KS: Formal analysis, Investigation, Validation, Writing – review & editing. PM: Funding acquisition, Project administration, Resources, Validation, Writing – review & editing. SD: Conceptualization, Funding acquisition, Methodology, Project administration, Resources, Supervision, Validation, Visualization, Writing – review & editing.
