# Supplementary material for: Genomic insights from Lactiplantibacillus plantarum BRD3A isolated from Atingba, a traditional fermented rice-based beverage and analysis of its potential for probiotic and antimicrobial activity against Methicillin-resistant Staphylococcus aureus
Source: Front Microbiol. 2024 Mar 27;15:1357818. doi: 10.3389/fmicb.2024.1357818 (PMC11019378; doi:10.3389/fmicb.2024.1357818)
Supplement: Supplementary file 1 [file Table_1.doc]

**SUPPLEMENTARY: Table** 1. Prediction of biosynthetic gene clusters of bacteriocin by BAGEL 4 Software.

| **Name** | **Function** | **Motifs** |
| --- | --- | --- |
| orf00001 |  |  |
| orf00003 |  |  |
| orf00004 |  |  |
| orf00006 |  |  |
| orf00008 | Galactoside O-acetyltransferase OS=Lactococcus lactis subsp. lactis (strain IL1403) OX=272623 GN=lacA PE=3 SV=1 |  |
| orf00011 | Quinolone resistance protein NorA OS=Staphylococcus aureus (strain MRSA252) OX=282458 GN=norA PE=3 SV=1 |  |
| orf00012 | 5-amino-6-(5-phospho-D-ribitylamino)uracil phosphatase YbjI OS=Escherichia coli (strain K12) OX=83333 GN=ybjI PE=1 SV=3 |  |
| orf00015 | HTH-type transcriptional activator RhaR OS=Salmonella arizonae (strain ATCC BAA-731 / CDC346-86 / RSK2980) OX=41514 GN=rhaR PE=3 SV=1 |  |
| orf00016 | Sugar phosphatase YidA OS=Shigella flexneri OX=623 GN=yidA PE=3 SV=1 |  |
| orf00018 | Branched-chain amino acid transport system carrier protein OS=Lactococcus lactis subsp. cremoris (strain MG1363) OX=416870 GN=brnQ PE=3 SV=1 |  |
| orf00021 | Na(+)/H(+) antiporter OS=Enterococcus hirae OX=1354 GN=napA PE=1 SV=1 |  |
| orf00024 | ComC; Lactococcin; Bacteriocin_IIc; | PF03047; PF04369; PF10439; |
| **97.2;Enterocin_X_chain_beta** | **ComC; Lactococcin; Bacteriocin_IIc; 97.2;Enterocin_X_chain_beta** | **PF03047; PF04369; PF10439;** |
| orf00026 |  |  |
| orf00029 |  |  |
| orf00031 | Putative bacteriocin Immunity protein |  |
| orf00032 | Bacteriocin_IIc; | PF10439; |
| orf00034 |  |  |
| orf00035 | Bacteriocin production related histidine kinase |  |
| orf00036 | response regulator PlnD | PF00072 |
| orf00040 | P71468_LACPL PlnI (Immunity protein PlnI, membrane-bound protease CAAX family) |  |
| **171.2;Plantaricin_F** | **ggmotif; Lactococcin; Bacteriocin_IIc; 171.2;Plantaricin_F** | **; PF04369; PF10439;** |
| **170.2;Plantaricin_E** | **170.2;Plantaricin_E** |  |
| LanT | Bacteriocin ABC-transporter, ATP-binding and permease protein PlnG | PF00005;PF03412 |
| HlyD | Accessory factor for ABC-transporter PlnH | PF13437 |
| orf00050 | PlnS |  |
| orf00051 |  |  |
| orf00053 | PlnS |  |
| orf00054 | PlnS |  |
| orf00055 |  |  |
| orf00058 | DNA helicase IV OS=Bacillus subtilis (strain 168) OX=224308 GN=helD PE=1 SV=1 |  |

**Table 2**: Prediction of gene clusters for RiPP and secondary metabolites

| **Contigs** |  | **Reference** | **Similarity score** | **Type** | **Compound(s)** | ***Organism*** |
| --- | --- | --- | --- | --- | --- | --- |
| 2.1 | T3PKS | [BGC0002674](https://mibig.secondarymetabolites.org/repository/BGC0002674/index.html#r1c1) | 0.23 | RiPP | enterocin F4-9 | *Enterococcus faecalis* |
|  | | [BGC0000286](https://mibig.secondarymetabolites.org/repository/BGC0000286/index.html#r1c1) | 0.23 | Polyketide | Viguiepinol | *Streptomyces sp. KO-3988* |
| [BGC0001387](https://mibig.secondarymetabolites.org/repository/BGC0001387/index.html#r1c1) | 0.22 | Other | Nucleocidin | *Streptomyces calvus* |
| [BGC0002561](https://mibig.secondarymetabolites.org/repository/BGC0002561/index.html#r1c1) | 0.19 | Alkaloid | phenazine SA, phenazine SB, phenazine SC | *Streptomyces sp.* |
| [BGC0002435](https://mibig.secondarymetabolites.org/repository/BGC0002435/index.html#r1c1) | 0.18 | Other | coformycin, aristeromycin | *Micromonospora haikouensis* |
| [BGC0001651](https://mibig.secondarymetabolites.org/repository/BGC0001651/index.html#r1c1) | 0.17 | Other | legionaminic acid | *Tannerella forsythia 92A2* |
| [BGC0000916](https://mibig.secondarymetabolites.org/repository/BGC0000916/index.html#r1c1) | 0.17 | Other | molybdenum cofactor | *Staphylococcus carnosus* |
| [BGC0001940](https://mibig.secondarymetabolites.org/repository/BGC0001940/index.html#r1c1) | 0.17 | Polyketide | pyxidicycline A, pyxidicycline B | *Pyxidicoccus fallax* |
| [BGC0002116](https://mibig.secondarymetabolites.org/repository/BGC0002116/index.html#r1c1) | 0.17 | RiPP | darobactin A | *Photorhabdus khanii HGB 1456* |
| [BGC0002699](https://mibig.secondarymetabolites.org/repository/BGC0002699/index.html#r1c1) | 0.16 | RiPP | nostolysamide A, nostolysamide B | *Nostoc punctiforme PCC 73102* |
| 2.2 | RiPP | [BGC0000617](https://mibig.secondarymetabolites.org/repository/BGC0000617/index.html#r1c1) | 0.22 | RiPP | coagulin | *Bacillus coagulans* |
|  | | [BGC0002585](https://mibig.secondarymetabolites.org/repository/BGC0002585/index.html#r1c1) | 0.21 | Other | ubericin K | *Streptococcus uberis* |
| [BGC0000589](https://mibig.secondarymetabolites.org/repository/BGC0000589/index.html#r1c1) | 0.2 | RiPP | microcin M | *Escherichia coli Nissle 1917* |
| [BGC0000586](https://mibig.secondarymetabolites.org/repository/BGC0000586/index.html#r1c1) | 0.19 | RiPP | microcin E492 | *Klebsiella pneumoniae RYC492* |
| [BGC0000619](https://mibig.secondarymetabolites.org/repository/BGC0000619/index.html#r1c1) | 0.17 | RiPP | gassericin T | *Lactobacillus gasseri* |
| [BGC0001602](https://mibig.secondarymetabolites.org/repository/BGC0001602/index.html#r1c1) | 0.17 | RiPP | gassericin-T | *Lactobacillus gasseri* |
| [BGC0001388](https://mibig.secondarymetabolites.org/repository/BGC0001388/index.html#r1c1) | 0.17 | RiPP | gassericin E | *Lactobacillus gasseri* |
| [BGC0001407](https://mibig.secondarymetabolites.org/repository/BGC0001407/index.html#r1c1) | 0.16 | RiPP | bicereucin | *Bacillus cereus SJ1* |
| [BGC0000590](https://mibig.secondarymetabolites.org/repository/BGC0000590/index.html#r1c1) | 0.16 | RiPP | microcin N | *Escherichia coli* |
| [BGC0001862](https://mibig.secondarymetabolites.org/repository/BGC0001862/index.html#r1c1) | 0.16 | RiPP | pallidocin | *Aeribacillus pallidus* |
| 13.1 | Terpene | [BGC0000647](https://mibig.secondarymetabolites.org/repository/BGC0000647/index.html#r1c1) | 0.45 | Terpene | carotenoid | *Rhodobacter sphaeroides* |
|  | | [BGC0000650](https://mibig.secondarymetabolites.org/repository/BGC0000650/index.html#r1c1) | 0.32 | Terpene | carotenoid | *Algoriphagus sp. KK10202C* |
| [BGC0000648](https://mibig.secondarymetabolites.org/repository/BGC0000648/index.html#r1c1) | 0.23 | Terpene | carotenoid | *Myxococcus xanthus* |
| [BGC0000656](https://mibig.secondarymetabolites.org/repository/BGC0000656/index.html#r1c1) | 0.23 | Terpene | zeaxanthin | *Xanthobacter autotrophicus Py2* |
| [BGC0000637](https://mibig.secondarymetabolites.org/repository/BGC0000637/index.html#r1c1) | 0.23 | Terpene | carotenoid | *Corynebacterium glutamicum* |
| [BGC0001227](https://mibig.secondarymetabolites.org/repository/BGC0001227/index.html#r1c1) | 0.21 | Terpene | isorenieratene | *Streptomyces collinus Tu 365* |
| [BGC0000633](https://mibig.secondarymetabolites.org/repository/BGC0000633/index.html#r1c1) | 0.21 | Terpene | carotenoid | *Streptomyces avermitilis* |
| [BGC0000640](https://mibig.secondarymetabolites.org/repository/BGC0000640/index.html#r1c1) | 0.2 | Terpene | carotenoid | *Enterobacteriaceae bacterium DC404* |
| [BGC0000636](https://mibig.secondarymetabolites.org/repository/BGC0000636/index.html#r1c1) | 0.2 | Terpene | carotenoid | *Brevibacterium linens* |
| [BGC0000630](https://mibig.secondarymetabolites.org/repository/BGC0000630/index.html#r1c1) | 0.2 | Terpene | (2R,3S,3'S)-2-hydroxyastaxanthin | *Paracoccus haeundaensis* |
| 13.2 | Autoinducer lactone cyclic | [BGC0000249](https://mibig.secondarymetabolites.org/repository/BGC0000249/index.html#r1c1) | 0.2 | Polyketide | nogalamycin | *Streptomyces nogalater* |
|  | | [BGC0001291](https://mibig.secondarymetabolites.org/repository/BGC0001291/index.html#r1c1) | 0.19 | RiPP | enterocin NKR-5-3B | *Enterococcus faecium* |
| [BGC0000217](https://mibig.secondarymetabolites.org/repository/BGC0000217/index.html#r1c1) | 0.16 | Polyketide | daunorubicin | *Streptomyces sp.* |
| [BGC0001880](https://mibig.secondarymetabolites.org/repository/BGC0001880/index.html#r1c1) | 0.16 | RiPP | scytodecamide | *Scytonema sp. UIC 10036* |
| [BGC0002579](https://mibig.secondarymetabolites.org/repository/BGC0002579/index.html#r1c1) | 0.15 | RiPP | carnobacteriocin XY | *Carnobacterium maltaromaticum* |
| [BGC0001198](https://mibig.secondarymetabolites.org/repository/BGC0001198/index.html#r1c1) | 0.13 | Terpene | (+)-O-methylkolavelool | *Herpetosiphon aurantiacus DSM 785* |
| [BGC0002362](https://mibig.secondarymetabolites.org/repository/BGC0002362/index.html#r1c1) | 0.13 | Polyketide | loseolamycin A1, loseolamycin A2 | *Micromonospora endolithica* |
| [BGC0000540](https://mibig.secondarymetabolites.org/repository/BGC0000540/index.html#r1c1) | 0.13 | RiPP | paenibacillin | *Paenibacillus polymyxa OSY-DF* |
| [BGC0002485](https://mibig.secondarymetabolites.org/repository/BGC0002485/index.html#r1c1) | 0.12 | RiPP, Alkaloid | muscoride A, muscoride B | *Desmonostoc sp. PCC 7906* |
| [BGC0001896](https://mibig.secondarymetabolites.org/repository/BGC0001896/index.html#r1c1) | 0.12 | Other | carbazomycin B | *Streptomyces luteoverticillatus* |
