# Supplementary figures and images for: Genomic insights from Lactiplantibacillus plantarum BRD3A isolated from Atingba, a traditional fermented rice-based beverage and analysis of its potential for probiotic and antimicrobial activity against Methicillin-resistant Staphylococcus aureus
Source: Front Microbiol. 2024 Mar 27;15:1357818. doi: 10.3389/fmicb.2024.1357818 (PMC11019378; doi:10.3389/fmicb.2024.1357818)

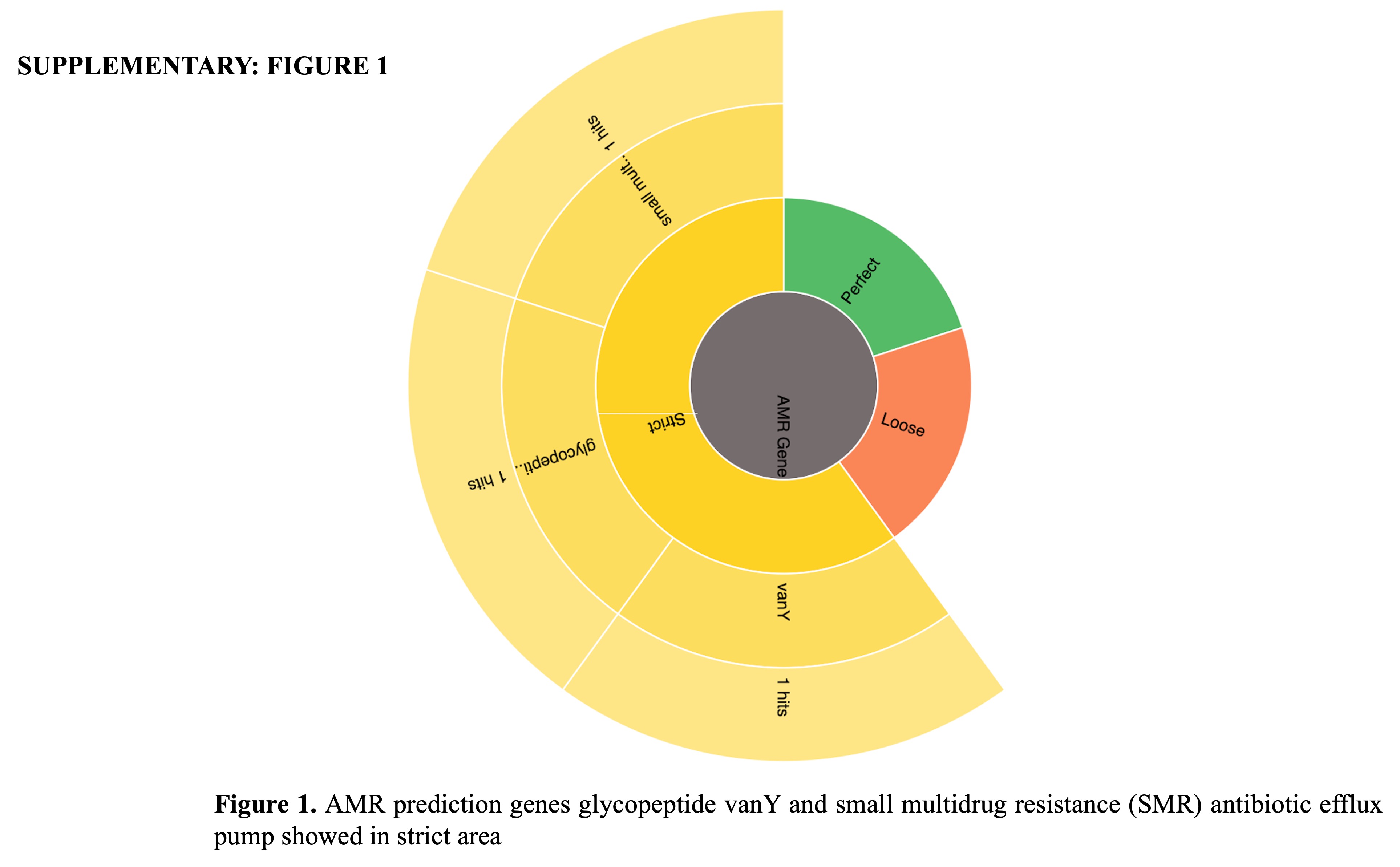

Supplement: Supplementary file 2 [file Image_1.JPEG]
